# Primary Mediastinal Pleural Hydatid Cyst Mimicking Tuberculous Pleuritis: A Case Report

**DOI:** 10.1002/ccr3.72156

**Published:** 2026-02-26

**Authors:** Grace Tannous, Omar Al Ayoubi, Alaa Senjab, Mohammad Abd Alrahman Saif, Mohammad hesso, Mohammad Akram Flioun, Bassam Darwish

**Affiliations:** ^1^ Faculty of Medicine Damascus University Damascus Syrian Arab Republic; ^2^ Thoracic Surgery Department Damascus University, Al Mouwasat University Hospital Damascus Syrian Arab Republic

**Keywords:** adenosine deaminase, case report, hydatid cyst, lymphocytic exudative pleural effusion, mediastinal pleura, tuberculosis

## Abstract

Hydatid disease, caused by various Echinococcus species, is endemic in developing countries and most commonly affects the liver and lungs. Approximately 7.4% of cases involve intrathoracic extrapulmonary locations, with pleural involvement usually resulting from rupture of adjacent hepatic or pulmonary cysts. However, primary pleural hydatid cysts are extremely rare, representing less than 1% of extrapulmonary cases, and may cause compressive symptoms depending on size and location. Diagnosis is guided by serologic testing and imaging, while surgical excision remains the mainstay of treatment. A 26‐year‐old Middle Eastern female presented to the emergency department with exertional dyspnea, right‐sided pleuritic chest pain, low‐grade fever, and night sweats. Physical examination revealed decreased breath sounds, dullness to percussion, and increased tactile fremitus on the right side, while imaging confirmed a moderate‐to‐large right pleural effusion with adhesions and fibrinous strands. Laboratory tests showed a lymphocytic exudative effusion with elevated adenosine deaminase, initially suggesting tuberculous pleuritis. However, negative cultures and positive anti‐Echinococcus antibodies redirected the diagnosis to hydatid disease. Video‐assisted thoracoscopic surgery (VATS) revealed dense adhesions and granulomatous inflammation on biopsy. Due to restricted lung expansion, right thoracotomy was performed, excising a cystic mass from the mediastinal pleura. Histopathology confirmed a hydatid cyst. The patient recovered well and was discharged on oral Albendazole. This case emphasizes the rarity of primary pleural hydatid cysts, the diagnostic challenges they pose, and the importance of considering parasitic infections in pleural effusions for timely surgical and medical management.

## Key Clinical Message

In endemic regions, primary pleural hydatid cysts, though extremely rare, may present with exudative pleural effusion and mimic tuberculous pleuritis. Failure to confirm tuberculosis should raise suspicion for parasitic etiologies. Timely recognition, surgical excision, and postoperative antiparasitic therapy are crucial for accurate diagnosis and optimal outcomes.

## 1 Introduction

Hydatid disease is a zoonotic infection caused by four distinct species of Echinococcus and remains endemic in many developing countries [1]. Humans acquire hydatid infection through ingestion of contaminated water or food, as well as direct contact with carnivorous animals [2]. It can occur in individuals of any age or gender, but it is more frequently observed in people between 20 and 40 years old [3]. Notably, pleural involvement in hydatid disease is uncommon and typically results from the rupture of a pulmonary or hepatic cyst into the pleural cavity, leading to secondary pleural hydatidosis [4]. However, primary echinococcal cysts involving the pleura are sporadic, representing less than 1% of all extrapulmonary hydatid disease cases [5]. Although hydatid cysts commonly present with a range of symptoms, they may also remain asymptomatic. Intrathoracic extrapulmonary cysts, in particular, can cause compressive symptoms affecting adjacent vital structures. Diagnosis relies on dermal tests, biochemical assays, and imaging [6]. Surgical treatment, including cystostomy or cystectomy with capitonnage, with or without pleural decortication, remains the mainstay of management [7].

This case highlights the rare presentation of an intrathoracic extrapulmonary hydatid cyst involving the mediastinal pleura, emphasizing the diagnostic challenge and the need for surgical intervention in such uncommon manifestations of hydatid disease.

## 2 Case History

A 26‐year‐old Middle Eastern female presented to the emergency department with exertional dyspnea and right‐sided pleuritic chest pain of one‐week duration, associated with low‐grade fever for 3 days and night sweats. She was a non‐smoker with no prior medical, surgical, allergic, or drug history. The patient did not report a clear history of contact with livestock or domestic animals. However, in endemic regions, exposure to Echinococcus could occur indirectly through contaminated food or water, even in the absence of identifiable risk factors. On admission, her vital signs were as follows: body temperature 38.2°C, heart rate 107 beats per minute, respiratory rate 24 breaths per minute, blood pressure 110/70 mmHg, and oxygen saturation 98% on room air. Physical examination revealed decreased breath sounds on the right side, increased tactile fremitus, and dullness to percussion over the right hemithorax. The rest of the physical examination was unremarkable.

## 3 Differential Diagnosis, Investigations and Treatment

**FIGURE 1:**
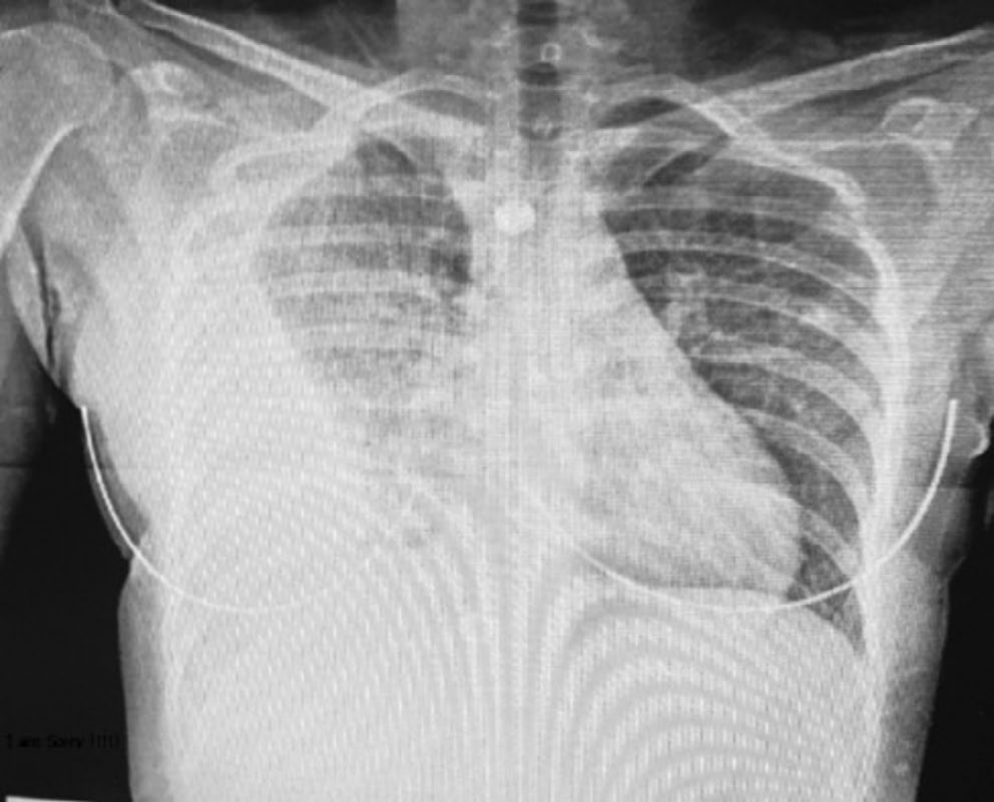
Preoperative posteroanterior chest radiograph showing complete right‐sided opacification with contralateral mediastinal shift.

**FIGURE 2:**
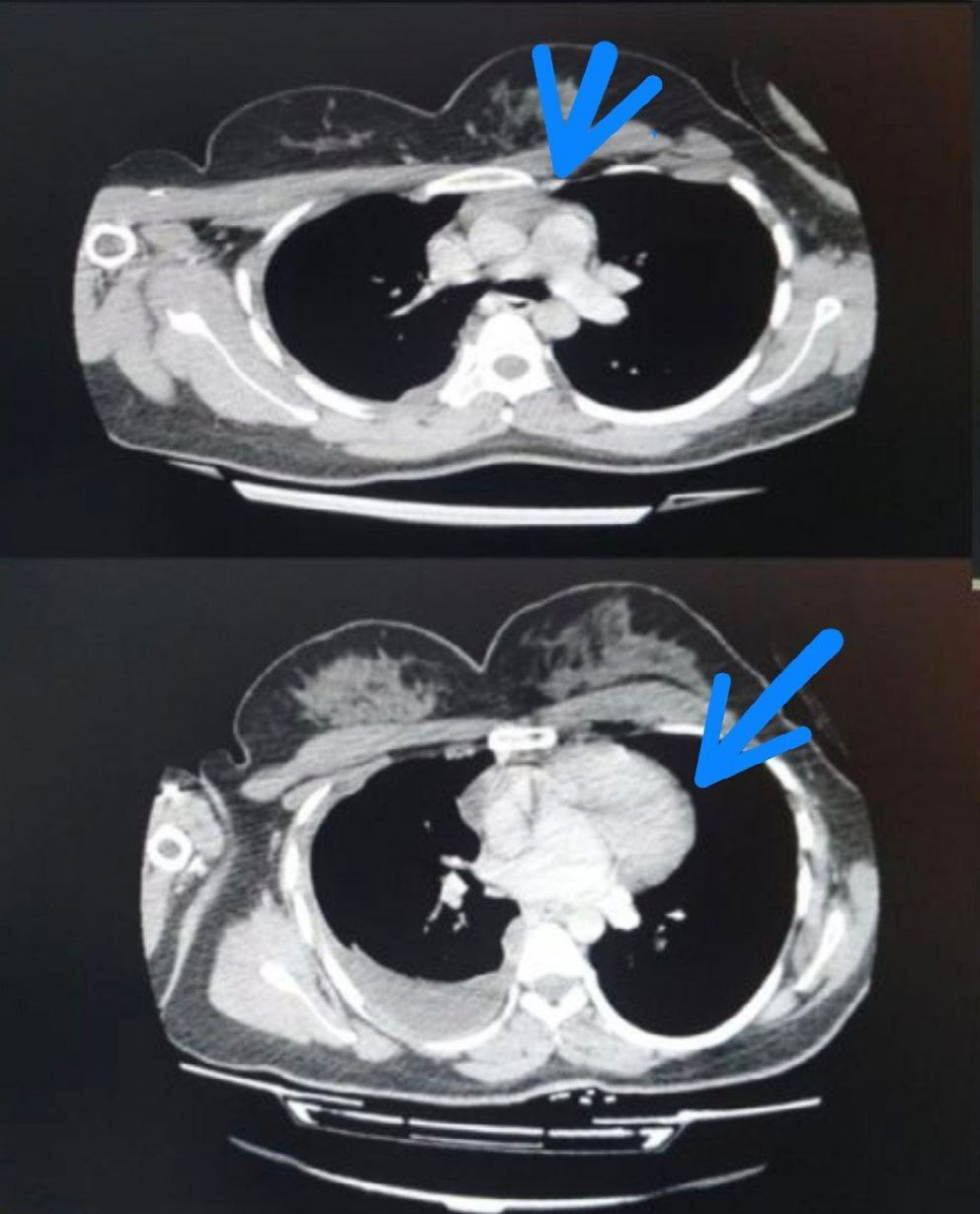
Contrast‐enhanced axial chest CT images (mediastinal window) show a well‐circumscribed, homogeneous, low‐attenuation pleural‐based lesion abutting the mediastinum, consistent with a cystic lesion. Additionally, a moderate associated pleural effusion is noted.

Following clinical evaluation, chest radiography revealed a right‐sided pleural effusion (Figure 1). A chest computed tomography (CT) scan demonstrated a well‐circumscribed, homogeneous, low‐attenuation pleural‐based lesion abutting the mediastinum, consistent with a cystic lesion. There was also moderate right pleural effusion with internal septations, fibrinous strands, and pleural adhesions (Figure 2). These findings differed from typical tuberculous pleuritis, which usually shows pleural thickening and effusion without a discrete cystic structure. No intact cyst was identifiable preoperatively, contributing to the initial diagnostic challenge.

Abdominal ultrasonography, chest imaging, and CT scan revealed no hepatic, peritoneal, or pulmonary lesions, which confirmed that the cyst involved only the mediastinal pleura. Therefore, this lesion can be considered a primary pleural hydatid cyst.

Laboratory investigations revealed a hemoglobin concentration of 13.1 g/dL, hematocrit 37%, white blood cell count of 10.2 × 10^3^ /μL (67% neutrophils, 27% lymphocytes, 2% eosinophils), and platelet count of 369 × 10^3^/μL. C‐reactive protein was elevated at 65 mg/L. Liver and kidney function tests were within normal limits, and coagulation parameters were normal.

A diagnostic and therapeutic thoracentesis was performed, and approximately one liter of pleural fluid was evacuated. The patient was started empirically on intravenous Ceftriaxone and Clindamycin. Pleural fluid analysis demonstrated an exudative lymphocytic effusion, with a total white blood cell count of 2156 cells/μL (84% lymphocytes), protein 5.7 g/dL, albumin 2.95 g/dL, glucose 20 mg/dL, pH 7.27, lactate dehydrogenase (LDH) 1724 U/L, and cholesterol 139 mg/dL. Notably, adenosine deaminase (ADA) was elevated at 49 U/L (normal < 24), initially favoring tuberculous pleuritis. However, pleural fluid cultures showed no bacterial growth, and tuberculosis was excluded by negative acid‐fast bacilli (AFB) smear and culture. Serological testing for hydatid disease was positive, with anti‐Echinococcus antibody titers > 1:160, redirecting the diagnostic consideration toward a parasitic etiology.

The differential diagnosis included tuberculous pleuritis, complicated parapneumonic effusion, empyema, pleural malignancy, lymphoma, and parasitic pleural effusion. Although the biochemical profile initially supported tuberculosis, negative microbiological studies and positive serology favored hydatid disease.

The patient was referred to thoracic surgery, where Video‐assisted thoracoscopic surgery (VATS) was initially performed. Intraoperatively, serous effusion, fibrinous material, and dense pleural adhesions were encountered. Multiple pleural biopsies were taken, and histopathological analysis revealed chronic inflammatory changes with non‐specific granulomatous reaction.

**FIGURE 3:**
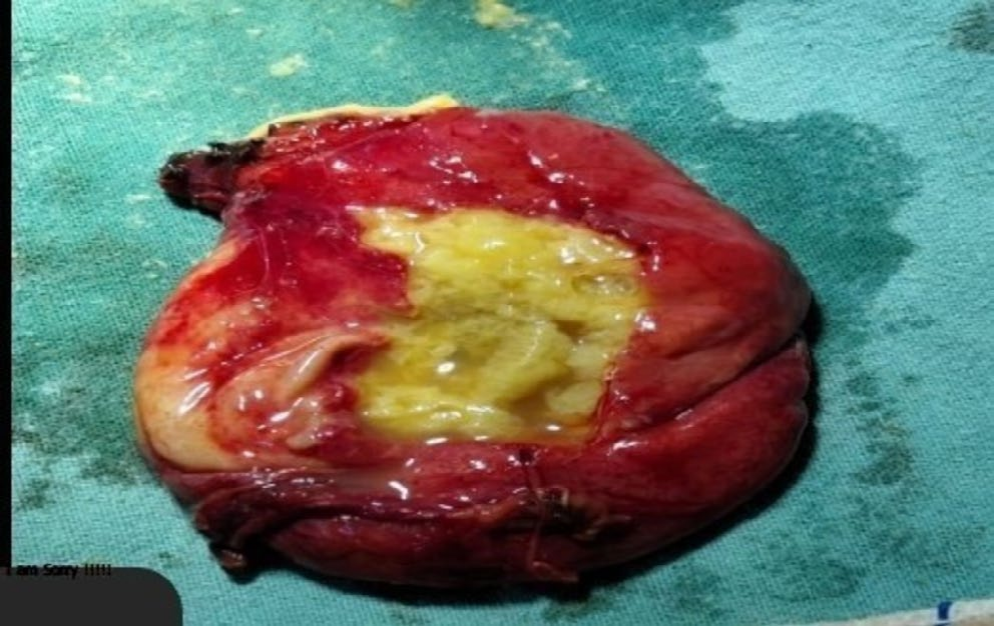
Intraoperative image showing the excised cystic lesion measuring approximately 6 × 5 cm. The cyst has a thick fibrous outer wall and contains yellowish gelatinous material, consistent with the gross appearance of a hydatid cyst.

**FIGURE 4:**
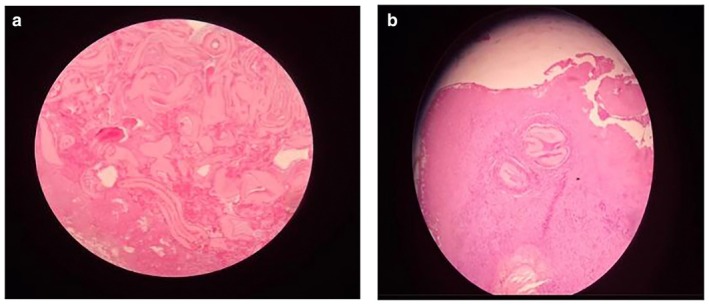
(a, b) Histopathological findings of hydatid disease (H&E stain). Representative low‐ and higher‐power micrographs show fragments of a thick acellular laminated hydatid membrane (ectocyst) surrounded by fibro‐granulation tissue with an eosinophil‐rich inflammatory infiltrate and foreign‐body type granulomatous reaction, with focal congestion/hemorrhage. No germinal layer or daughter cysts are identified in the examined sections.

Due to extensive pleural thickening and restricted lung expansion, a right posterolateral thoracotomy through the fifth intercostal space was performed. A thick fibrous peel covering the right lung was decorticated completely. A cystic lesion measuring 6 × 5 cm was excised from the mediastinal pleura (Figure 3). Microscopic examination revealed a thick acellular laminated membrane surrounded by granulation tissue and fibrosis, with dense inflammatory infiltrates including eosinophils and lymphocytes. A granulomatous reaction with areas of congestion and hemorrhage was also identified. Although no germinative layer or daughter cysts were observed, the presence of a characteristic laminated membrane with eosinophilic inflammatory response is sufficient to support the diagnosis of hydatid disease. No evidence of malignancy was found (Figure 4).

## 4 Conclusion and Results

**FIGURE 5:**
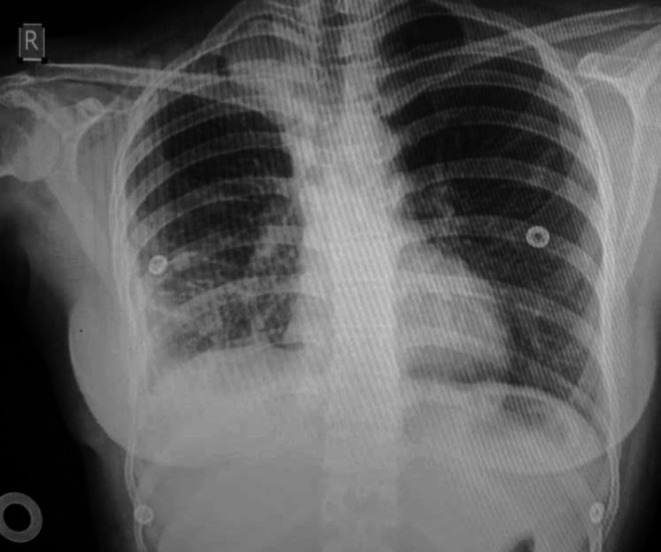
Postoperative chest radiograph (portable AP view) shows improved right lung expansion.

Final histopathological analysis confirmed the diagnosis of a hydatid cyst involving the mediastinal pleura. The patient's postoperative course was uneventful, and she was discharged 3 days after surgery in stable condition. Oral Albendazole 400 mg daily was prescribed for 6 months (Figure 5).

## 5 Discussion

Hydatid disease, caused by Echinococcus granulosus, is endemic in several Mediterranean countries and affects nearly one million people worldwide, according to the World Health Organization (WHO) [8]. Although hydatid cysts can develop in virtually any part of the body, they are most frequently found in the liver (75%) and the lungs (25%) [3, 7]. Intrathoracic extrapulmonary hydatid cysts are a rare clinical entity, accounting for approximately 7.4% of cases [9]. These cysts may involve various thoracic structures. About 55% located within the intrapulmonary fissures, 18% in the parietal pleura, 14% in the chest wall, and 4.5% each in the mediastinum and diaphragm [10]. Primary pleural hydatidosis is extremely rare, accounting for less than 1% of all hydatid disease cases, and can mimic other pleural pathologies [11]. This case is unique due to its isolated mediastinal pleura without pulmonary or hepatic involvement. It closely resembled tuberculous pleuritis biochemically and clinically, which created a significant diagnostic pitfall. Few cases in the literature report similar mediastinal pleural involvement. Elevated ADA with lymphocytic effusion in these cases has been noted as a diagnostic pitfall [11]. This case highlights the need to consider hydatid disease in unexplained pleural effusions in endemic regions. Most hydatid cysts are asymptomatic and incidentally discovered. Symptomatic patients may present with chest pain, cough, dyspnea, or reduced lung volume [12]. Our patient had exertional dyspnea, pleuritic chest pain, and systemic symptoms (fever, night sweats), which are consistent with the hydatid disease spectrum. The diagnostic challenge arose from the atypical cyst location and pleural fluid features mimicking tuberculosis: lymphocytic exudate, elevated ADA, low glucose, and high LDH [13, 14]. Elevated ADA levels are not specific to tuberculosis and may also be observed in parasitic pleural effusions. This increase reflects activation of T lymphocytes and macrophages during cell‐mediated immune responses within the pleural space [15]. Tuberculosis was excluded by negative pleural fluid cultures and the absence of acid‐fast bacilli on smear and culture. Positive serology for Echinococcus antibodies confirmed a parasitic etiology, highlighting the importance of immunodiagnostic tests for accurate diagnosis. Enzyme‐linked immunosorbent assay (ELISA), which can achieve up to 95% sensitivity in hydatid disease, confirmed the presence of anti‐Echinococcus antibodies and redirected the diagnostic approach toward hydatid cyst [16]. Imaging modalities such as chest CT, chest radiography, and abdominal ultrasonography contributed significantly to both diagnosis and surgical planning.

VATS is essential for the diagnostic evaluation of undiagnosed exudative pleural effusions, offering high accuracy through direct visualization and tissue biopsy [17]. In the present case, histopathological examination of the pleural biopsy revealed only chronic inflammation with non‐specific granulomas. However, intraoperative identification and excision of a cystic mass allowed the definitive diagnosis of a hydatid cyst as summarized in (Table 1).

**TABLE 1:** comparison between tuberculous pleuritis and hydatid (parasitic) pleural effusion.

Feature	Tuberculosis	Hydatid cyst (parasitic effusion)
ADA	↑↑ markedly elevated due to T‐lymphocyte activation [18, 19]	↑ (sometimes); non‐specific elevation related to T‐lymphocyte and macrophage activation [13, 15]
Glucose	↓ low pleural fluid glucose [14, 18]	↓ (variable); may be reduced in inflammatory parasitic effusions [11, 19]
LDH	↑ elevated LDH reflecting intense pleural inflammation [18]	↑ (variable); depends on degree of pleural inflammation [11, 13]
Lymphocytes	Lymphocytic predominance common [18]	Lymphocytic predominance frequently reported [11, 16]
Imaging	Pleural thickening and effusion without discrete cystic lesion [2, 18]	Cystic lesion, membranes, pleural adhesions, extrapulmonary masses [2, 7]
Serology	Positive TB tests (AFB smear/culture, Interferon‐Gamma Release Assay (IGRA)) [18]	Positive anti‐Echinococcus antibodies (ELISA) [16]

Despite surgical mortality rates of up to 4% in some series, surgery remains the treatment of choice for hydatid cysts when no contraindications exist [20]. Our patient underwent successful cyst excision via thoracotomy without complications, highlighting the safety and effectiveness of surgical management in such cases.

Postoperative antiparasitic therapy is essential to prevent recurrence. Albendazole, administered at a dose of 400 mg orally for at least 6 months, is the standard of care [20]. In our case, the patient received Albendazole postoperatively, with favorable clinical evolution and no early signs of recurrence.

## 6 Limitations

This case report has certain limitations. Due to the challenging economic conditions and limited healthcare resources in the local setting, routine health checkups and screening investigations, such as systematic cancer screening or advanced immunological testing, are not universally accessible. As a result, some investigations that might be performed in higher‐resource settings were not available in this case. Nevertheless, the diagnosis was established through a combination of clinical presentation, imaging, serological testing, intraoperative findings, and definitive histopathological confirmation, which together provided sufficient evidence to support the final diagnosis.

## 7 Conclusion

This case emphasizes the diagnostic challenge of primary pleural hydatid cysts due to their rarity, atypical location, and biochemical resemblance to TB. Clinicians in endemic regions should consider parasitic etiologies in exudative pleural effusions. A systematic approach combining biochemical, serological, imaging, and surgical data ensures accurate diagnosis. Early recognition and appropriate management are crucial to achieve favorable outcomes and prevent complications.

## Author Contributions

**Grace Tannous:** conceptualization, methodology, project administration, supervision, writing – original draft, writing – review and editing. **Omar Al Ayoubi:** data curation, methodology, software, writing – original draft, writing – review and editing. **Alaa Senjab:** conceptualization, methodology, software, visualization, writing – original draft, writing – review and editing. **Mohammad Abd Alrahman Saif:** investigation, validation, writing – review and editing. **Mohammad hesso:** investigation, methodology, resources, writing – review and editing. **Mohammad Akram Flioun:** investigation, methodology, resources, writing – review and editing. **Bassam Darwish:** conceptualization, formal analysis, investigation, methodology, project administration, supervision, writing – review and editing.

## Funding

The authors have nothing to report.

## Ethics Statement

Institutional Review Board (IRB) approval is not required for de‐identified single case reports or case histories, in accordance with institutional policies.

## Consent

Written informed consent was obtained from the patient for publication and any accompanying images. A copy of the written consent is available for review by the Editor‐in‐Chief of this journal on request.

## Conflicts of Interest

The authors declare no conflicts of interest.

## Data Availability

Data available on request from the authors.
